# Beyond *R*_0_: Demographic Models for Variability of Lifetime Reproductive Output

**DOI:** 10.1371/journal.pone.0020809

**Published:** 2011-06-29

**Authors:** Hal Caswell

**Affiliations:** 1 Biology Department, Woods Hole Oceanographic Institution, Woods Hole, Massachusetts, United States of America; 2 Max Planck Institute for Demographic Research, Rostock, Germany; University of California, Berkeley, United States of America

## Abstract

The net reproductive rate 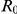 measures the expected lifetime reproductive output of an individual, and plays an important role in demography, ecology, evolution, and epidemiology. Well-established methods exist to calculate it from age- or stage-classified demographic data. As an expectation, 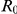 provides no information on variability; empirical measurements of lifetime reproduction universally show high levels of variability, and often positive skewness among individuals. This is often interpreted as evidence of heterogeneity, and thus of an opportunity for natural selection. However, variability provides evidence of heterogeneity only if it exceeds the level of variability to be expected in a cohort of identical individuals all experiencing the same vital rates. Such comparisons require a way to calculate the statistics of lifetime reproduction from demographic data. Here, a new approach is presented, using the theory of Markov chains with rewards, obtaining all the moments of the distribution of lifetime reproduction. The approach applies to age- or stage-classified models, to constant, periodic, or stochastic environments, and to any kind of reproductive schedule. As examples, I analyze data from six empirical studies, of a variety of animal and plant taxa (nematodes, polychaetes, humans, and several species of perennial plants).

## Introduction

The net reproductive rate 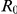 is a familiar concept in demography. It has three important properties [1]–[3]: it measures mean lifetime reproductive output, it is the population growth rate per generation (not per unit of time), and it is an indicator function for population growth, in that population growth is positive if and only if 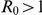. It is calculated from age-classified models as
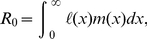
(1)where 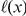 is surivorship to age 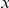 and 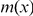 is fertility at age 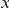 [4], and from stage classified models as
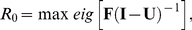
(2)where 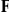 is a matrix of stage-specific fertilities and 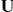 is a matrix giving transition probabilities of individuals among stages [1]–[3], [5]. In evolutionary biology, 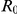 is sometimes used as a measure of fitness, although this works only under certain circumstances; e.g. [6]–[10]. In epidemiology, 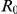 gives the expected number of secondary infections following the introduction of a single infectious individual into a susceptible population [11]–[13]. The infection can spread and produce an outbreak if and only if 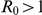.

The net reproductive rate, however, is an expectation. Measurements of lifetime reproduction invariably show variability – often large amounts of variability – among individuals. The distribution is often positively skewed, with a long tail of rare individuals producing more than the average number of offspring; e.g., many examples in [14], [15]. Variability in lifetime reproduction is an important demographic property [16], with many consequences. Skewness among individuals in disease transmission affects the likelihood and severity of disease outbreaks [17]. Variance in lifetime reproductive output is one of the determinants of the genetic effective population size [18], [19]. The observed variability and skewness of lifetime reproduction is sometimes interpreted as evidence of heterogeneity among individuals, or as part of a strategy in which dominant individuals control reproduction by subordinate individuals [20], [21]. If such heterogeneity exists and has a genetic basis, the resulting variability in lifetime reproduction provides an opportunity for selection; the variance in lifetime reproduction is part of one measure of the opportunity for selection [22].

However, variability in lifetime reproduction is to be expected even in the absence of heterogeneity. One source of variability is stochastic variation among individuals in the pathways they take through the life cycle (“individual stochasticity” in the usage of Caswell [3], “dynamic heterogeneity” in the usage of Tuljapurkar and Steiner [23], [24]). A cohort of identical individuals, experiencing identical vital rates at every stage, will differ in how long they live and how long they spend in each stage [3]. A second source of variation is within-stage variation in reproduction. A cohort of identical individuals, in the same stage, experiencing the same probability distribution of stage-specific reproduction, will differ in how many offspring they produce.

Thus, variability in lifetime reproductive output is evidence for heterogeneity only if it exceeds the baseline level created when a set of identical vital rates are applied to a cohort of identical individuals. Such comparisons require a way to calculate that baseline, as (1) and (2) do for the expectation. An limited approach for age-classified populations was presented by Barrowclough and Rockwell [19], [25]. A partial solution was provided by Caswell [3], [26] for the special case of life cycles that contain a “breeding” stage; e.g., [27]–[33]. Steiner and Tuljapurkar [34] have independently analyzed variability in lifetime reproduction, for some special cases of the models to be analyzed here, using different methods and emphasizing the importance of distinguishing variance due to individual stochasticity form that due to genetic variation.

In this paper, I derive, for the first time, a general and tractable calculation of all the moments of lifetime reproductive output, for stage- or age-classified populations, for arbitrary distributions of stage-specific reproduction, in constant, periodic, and stochastic environments. The calculations use a mathematical framework (Markov chains with rewards) that is new to population biology, but which has great potential applications to questions in addition to lifetime reproductive output. In the remainder of this *Introduction*, I present the mathematical framework and how to adapt it to the problem of lifetime reproduction. In the section *Analysis*, I formally prove the results on moments of lifetime reward, in both constant and time-varying environments. In the section *Case Studies*, I analyze a series of examples, ranging from laboratory studies of genetically identical individuals in constant conditions to field studies of genetically heterogeneous populations in stochastic environments. I conclude with a Discussion.

Notation: Matrices are denoted by upper-case bold symbols (e.g., 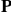), vectors by lower-case bold symbols (e.g., 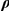). Some block-structured matrices are denoted by, e.g., 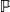. Vectors are column vectors by default. The transpose of 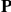 is 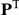. The vector 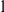 is a vector of ones, 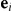 is a vector with a 1 in the 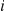
th entry and zeros elsewhere. The diagonal matrix with the vector 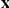 on the diagonal and zeros elsewhere is denoted 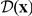. The expected value is denoted by 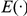. The Hadamard, or element-by-element, product of matrices 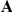 and 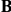 is denoted by 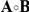. The Kronecker product is denoted by 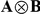.

### Markov chains with rewards

I propose to analyze lifetime reproductive output using the theory of *Markov chains with rewards* [35]–[37]. These models use a Markov chain to describe the dynamics of a system, and associate a reward with each possible transition among the states of the Markov chain. Rewards accumulate as the system moves from state to state, and the goal is to compute the properties of this accumulated reward. Markov chains with rewards are used to analyze the reliability of industrial and engineering systems [38], [39]. In demography, the Markov chain describes transitions among life cycle stages, with death as an absorbing state [2], [3], [26], [28], [29], [40], [41]. The transition matrix of this absorbing chain is
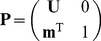
(3)where 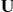 is the transient matrix (dimension 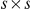) and 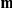 a vector of mortality rates. I will assume throughout that the dominant eigenvalue of 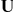 is less than 1, so that an individual beginning in any transient state will eventually be absorbed (i.e., will eventually die) with probability 1.

In a Markov chain with rewards, an individual moving from state 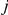 to state 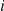 collects a reward 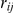. In the application here, the reward corresponds to reproduction. Later I will discuss other uses for the approach. Markov chains with rewards were introduced by Howard [35] to analyze Markov decision processes. In his development, the reward 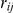 was a fixed quantity. Here, however, I will consider the 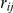 to be random variables with specified statistical properties [42]. Fixed rewards are included as a special case.

### Reproduction as a reward

In most matrix population models, reproduction between 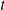 and 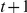 is a function of the stage at time 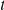, independent of the stage to which the individual moves at 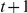. If this is so, the 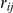 will depend only on 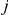, but this restriction can be relaxed. The exceptions to this rule are models with explicit reproductive stages, in which reproduction is associated with the transition into a reproductive state; e.g., [29]. In these cases, 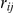 will depend explicitly on both 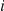 and 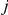. In demographic models, it is also the case that the dead do not reproduce (I know of no exceptions, but the recent literature on the population biology of zombies [43] may yet provide one). Thus 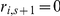, for all 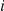, in the models here.

## Analysis

As an individual moves through the stages of the life cycle, it accumulates rewards. The goal of the analysis is to calculate the statistical properties (mean, variance, skewness) of the accumulated lifetime reward. The solution to this problem is provided by an simple set of recurrence relations.

Define 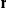 as the vector (dimension 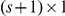) of accumulated rewards as a function of the initial stage of the individual. The vector of 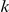
th moments of the entries of 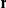 is denoted 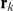, where
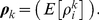
(4)The rewards 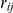 are random variables. The matrix of the 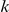
th moments of the 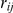 is denoted 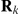:
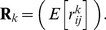
(5)Notation alert: The subscripts on the vectors 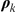 and the matrices 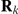 denote the order of the moments. When referring to the entries of the vector or the matrix, subscripts refer to the location in the matrix and the order of the moments migrates to become a parenthetical superscript. That is, the 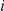
th entry of 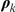 is 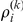 and the 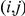 entry of 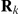 is 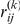.

The calculation of the accumulated rewards proceeds in the “backwards” fashion familiar from dynamic programming; e.g., [35], [44]. Choose some terminal time 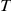, define 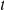 as the time remaining until this terminal time, and let 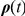 be the reward yet to be accumulated at 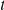. At the terminal time, no more rewards will be accumulated, so 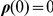.

Consider an individual in state 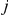 with 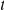 steps remaining to the terminal time. If this individual makes a transition from 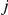 to 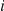, it will receive a reward 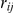. After the transition, the individual is in stage 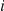 and has 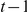 time steps remaining to the terminal time. Thus the conditional expectation of the reward in stage 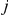, given the transition from 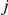 to 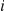, is
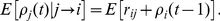
(6)The unconditional expectation of 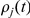 is
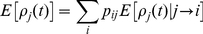
(7)

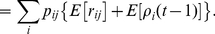
(8)Writing this in matrix form gives the recursion relation for the first moment of rewards
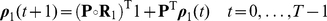
(9)with initial condition 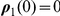, where 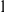 is a vector of ones [35, Eq. 2.5].

The combination of the assumptions that 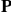 has the structure (3) and that 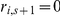 for all 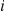 means that every individual will eventually be absorbed in a state in which future rewards are zero; thus 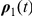 will converge to a limit as 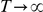; this limit is the expectation of lifetime rewards calculated over the entire lifetime of every individual. See the section *Discussion* for discounting necessary to calculate asymptotic rewards in ergodic Markov chains, when this eventual end to accumulation does not hold.

The main result of this paper is the following set of recurrence relations for the higher-order moments of accumulated rewards.

### Calculating the moments of lifetime rewards

**Proposition 1** Let 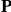 be the transition matrix of the Markov chain, let 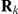 be the matrix of 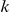
th moments of the transition-specific rewards, and let 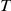 denote the terminal time. The first three moments of the accumulated reward satisfy
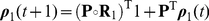
(10)

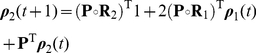
(11)

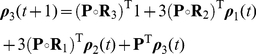
(12)for 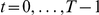, with 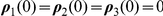. In general, the 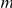
th moments of accumulated rewards are given by
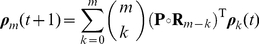
(13)with 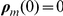.

#### Derivation

Equation (10) for the first moment is derived as (9). The conditional second moment of an individual in stage 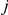, given a transition from 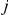 to 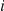, satisfies
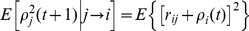
(14)

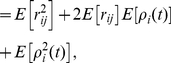
(15)because 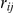 and 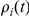 are independent. The unconditional second moments are
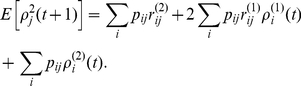
(16)Rewriting this in matrix form gives (11).

The conditional third moment of accumulated reward, for an individual in stage 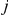, is
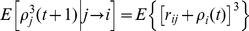
(17)

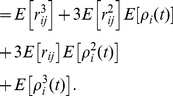
(18)The unconditional moments are
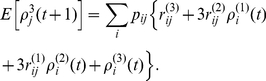
(19)Rewriting this in matrix form gives (12).

In general, expanding the conditional expectation of the 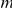
th moment gives
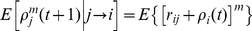
(20)

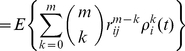
(21)

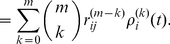
(22)The unconditional expectation is then
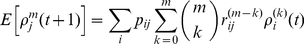
(23)which, in matrix form, becomes (13).

The first moment 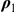 gives the mean lifetime reproductive output. This will often (but not always) be equivalent to 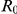 calculated from the Cushing-Zhou formula (2). See the Discussion for an exploration of the relationship between the two. The variance, standard deviation, coefficient of variation, and skewness of lifetime reproductive output are calculated from the moment vectors
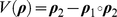
(24)

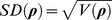
(25)

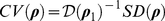
(26)

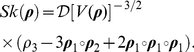
(27)The variance is useful because it can be partitioned additively among sources. The standard deviation cannot be partitioned in this way, but it has the advantage of appearing in the same units as 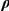. The CV scales the standard deviation relative to the mean, and hence is dimensionless. The CV is also the square root of Crow's [22] index of the opportunity for selection; this provides a upper bound on the rate of increase of mean fitness, if fitness is measured by lifetime reproduction and all the variance in reproduction is genetic. Finally, the skewness, which is dimensionless, measures the symmetry of the distribution of rewards. Positive skewness implies a long tail of positive values, and vice versa.

Several authors in the widely scattered literature on Markov chains with rewards have addressed the variance of accumulated rewards. Sladky and van Dijk [45], [46] have given results for discrete- and continuous-time chains with fixed rewards. Benito [42] provides variances for discrete chains with random rewards; my proof of Proposition 1 follows his approach.

### Distributions of stage-specific rewards

The statistics of lifetime reproduction depend, in equations (10)–(12), on the moments of the stage-specific rewards 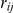. These moments can be obtained in several ways.

1. Empirical measurement. Given stage-specific individual data on reproductive output, the moment matrices 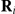 can be calculated directly. Such data are often collected, but the moments other than the mean are seldom published.
  If the empirical moments are not available, they can be estimated by applying a statistical model, such as:
2. The Poisson model. Given a mean reproductive output 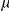, the Poisson distribution [47] describes a random distribution of reproduction among individuals, and leads to
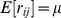
(28)

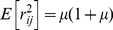
(29)

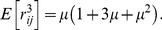
(30)
3. The Bernoulli model. In species that produce at most a single offspring, the mean reproductive output 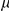 is equal to the probability of reproducing, and the number of offspring has a Bernoulli distribution with
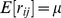
(31)

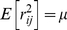
(32)

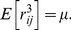
(33)
4. The fixed reward model. If stage-specific rewards were fixed, instead of random variables, then every individual would produce the same number of offspring, so that
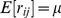
(34)

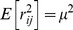
(35)

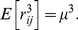
(36)Comparing the fixed reward model with one of the random reward models makes it possible to partition variance in lifetime reproduction into components due to variability in stage-specific rewards and to variability in the visits by individuals to the various stages.

### Lifetime reproduction in variable environments

In a variable environment, both the Markov transition matrix 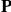 and the reward matrices 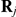 may change over time. The variation may be periodic, deterministic but aperiodic, or stochastic. The distribution of accumulated rewards will depend on the pathways followed by individuals through the life cycle, which in turn will depend on the trajectory followed by the environment through its set of states. As a result, the statistics of lifetime reproduction in a variable environment depend on both the initial stage of the individual and the initial state of the environment. For example, the lifetime reproduction of a seedling that germinates in early spring will be very different from that of seedling germinating in late summer; see [3], [26], [48] for discussions of the effect of starting state in the analysis of survival and longevity.

The demographic net reproductive rate 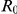 can be calculated in periodic environments by extending the Cushing-Zhou approach to periodic matrix products [3], [30]; for a more detailed analysis see [49]. (I note that [1] has been cited by many, including me, to Cushing and Yicang, an unfortunate confusion of the family and personal names of Zhou Yicang. I regret contributing to this confusion.) Here, I apply Proposition 1 to variable environments by creating a Markov chain in which individuals are jointly classified by life cycle stage and environmental state [3], [26]. This Markov chain is based on the vec-permutation model introduced by Hunter and Caswell [50] for individuals classified by stage and location.

I assume a finite number 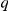 discrete environmental states. These could represent, e.g., seasons of the year, stages of recovery from fire, or years in an observed historical sequence. Define the reward vector for the joint process as
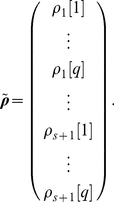
(37)That is, the first 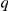 entries of 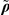 contain the rewards for stage 1 in each of the 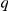 environments, and so on.

Associated with each environmental state is a transition matrix and a set of reward moment matrices:
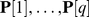
(38)

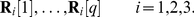
(39)The movement of the environment among its states is governed by a 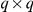 transition matrix 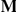. If the environment is stochastic, 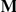 is a column-stochastic Markov chain transition matrix. If the environment is periodic, numbering the environmental states in order of occurrence makes 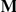 a circulant matrix of the form (for the case when 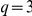)
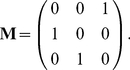
(40)If the environment is deterministic but aperiodic, moving through a specified sequence of states, then by numbering the states in the order in which they occur 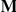 can be written
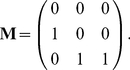
(41)The 1 in the 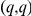 entry is required to provide an end state for the environmental sequence.

Starting at some time 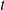, an individual makes a demographic transition according to 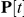 and collects rewards according to 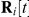, after which the environment changes to its next state according to 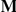 and the process repeats. Rewards are based on the demographic transition, and not on the environmental change; this assumption is implicit in all time-varying demographic models of which I am aware.

To model this process, define block matrices for demographic transitions
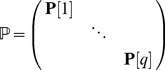
(42)

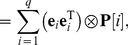
(43)environmental transitions,
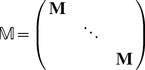
(44)

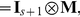
(45)and rewards
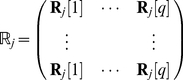
(46)

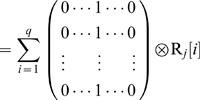
(47)

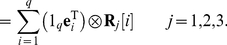
(48)Here, 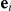 is the 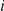
th unit vector, 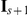 is the identity matrix of dimension 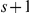, and 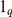 is a vector of ones of dimension 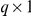.

In terms of these block matrices, the transition matrix and reward matrices are
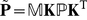
(49)

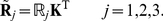
(50)where 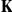 is the vec-permutation matrix of order 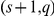, given by
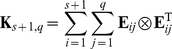
(51)with 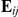 is a 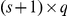 matrix with a 1 in the 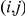 entry and zeros elsewhere [51]. This permutation matrix rearranges the entries of the probability vector so that block-diagonal forms can be used for the matrices 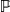 and 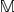 [50].

Since 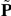 defines a Markov chain, Proposition 1 can be applied directly. The resulting reward vectors 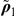, 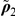, and 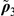 give the moments of lifetime reward as a function of the initial stage of the individual and the initial state of the environment, arranged as in (37). These moment vectors can be averaged over the distribution of states, thereby obtaining a summary measure of accumulated rewards as a function of initial stage alone. In a stochastic environment, the stationary probability distribution of environmental states is given by the vector 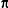 satisfying
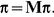
(52)The average, over the stationary distribution 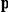, of the 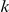
th moments of the lifetime reward, as a function of initial life cycle stage, is
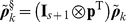
(53)cf. notation in [3]. Variances, standard deviations, skewness, and other statistics can be calculated from the moment vectors 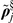 .

## Case Studies

Novel demographic calculations acquire much of their power from comparative studies. As a step in that direction, I present here several examples of calculation of the statistics of lifetime reproduction. The studies were selected to provide examples of different life histories, study designs, and types of data. The first example is a laboratory study of three genotypes of the nematode *Caenorhabditis elegans*. The second example is a laboratory study of the estuarine polychaete *Streblospio benedictii* under four pollutant exposure conditions. The third example is a historical sequence of vital rates for the human population of Sweden from 1891 to 2007. These three studies are age-classified, but the distribution of rewards differs, with *C. elegans* and *S. benedicti* producing large clutches, while humans are (to a good approximation) monovular.

The fourth example is a stage-structured analysis of a plant, *Trillium grandiflorum*, in an experiment in which pollination manipulations were applied to alter reproductive output. The final two examples explore the effects of temporal variation in the environment. The first is a periodic model for seasonal variation in the perennial plant *Lobularia maritima*. Two kinds of reproduction (seeds and seedlings) appear in this model; the approach here permits analysis of lifetime reproductive output for each type of offspring separately. The final example is an analysis of the threatened perennial plant *Lomatium bradshawii* in a stochastic fire environment.

These examples include age-classified and stage-classified life cycles, laboratory and field studies, and constant and variable environments, and include comparisons among experimental conditions, over time, or as a response to environmental fluctuations. In some cases, data are available on the moments of stage-specific reproductive output. In other cases, only the mean is available, and the higher moments must be obtained from a model. I intend them not as a complete survey of patterns, but as examples of the kinds of data that investigators using these methods might want to explore.

### Case study 1: Longevity mutants in *Caenorhabditis elegans*

The nematode *C. elegans* is widely used as a model organism for studies of genetics, development, aging, and behavior. A number of mutations have been identified that have dramatic effects on longevity, through a variety of developmental pathways [52], [53]. These mutations affect lifetime reproductive output both through their effects on longevity, but also from pleiotropic effects on fertility.

Chen et al. [54] carried out laboratory life table studies of three genetic strains of *C. elegans*: the standard laboratory strain *N2* and two well-studied longevity mutants, *clk-1* and *daf-2*. The *clk-1* gene affects metabolic activity and extends longevity, perhaps by reducing production of reactive oxygen. The *daf-2* gene also extends longevity; it codes for an insulin-like growth factor (IGF-I) receptor, which is part of a signaling cascade that influences life span [53].

Under laboratory conditions, life expectancies for the three strains were 14.3 days for *N2*, 18.3 days for *clk-1*, and 30.3 days for *daf-2*. In spite of their greater longevity, however, the *clk-1* and *daf-2* mutants had significantly lower fitness due to associated reductions in early fertility [54].

*C. elegans* is a self-fertilizing hermaphrodite. Laboratory cultures are homozygous and genetically homogenous, and are grown under carefully controlled conditions to minimize environmental differences among individuals. To the extent that heterogeneity is supposed to have a genetic basis, laboratory populations of *C. elegans* should exhibit as little heterogeneity as possible.

#### Study design

Individual survival and reproductive data were collected on cohorts of nematodes in laboratory culture. Because the study collected individual cohort data, the observed distributions of age-specific rewards and of lifetime reproductive output are available.

#### The demographic model

Demography was described with an age-classified projection matrix with a projection interval of 1 day; see [54] for details.

#### Rewards

Reproduction was measured as egg production and was recorded for each individual on each day, hence
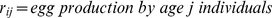
(54)Rewards were described by their empirical moments, by the Poisson model, and by the fixed rewards model.

#### Results

**Figure 1:**
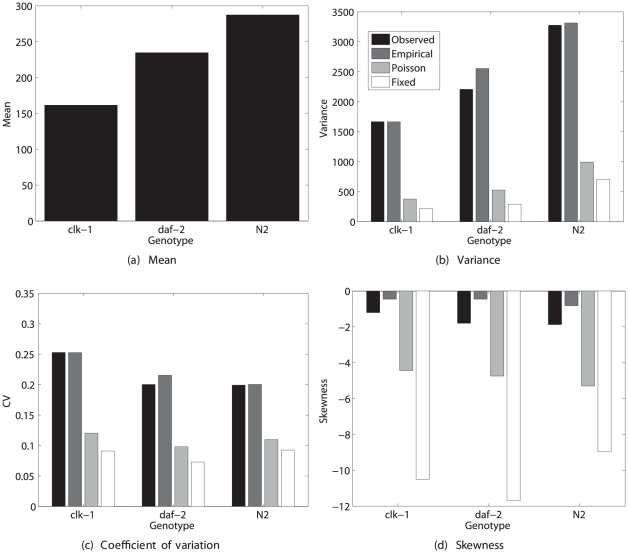
The statistics (mean, variance, coefficient of variation, and skewness) of lifetime reproductive output of the nematode *Caenorhabditis elegans*. Results are shown for two longevity mutants (*clk-1* and *daf-2*) and the standard laboratory strain (*N2*). The observed statistics of lifetime reproduction are compared with values calculated from the empirical moments, the Poisson reward model, and the fixed reward model. Based on data from [54].

The statistics of lifetime reproduction calculated from the demographic model are shown in Figure 1. The *clk-1* and *daf-2* genotypes exhibit reduced mean lifetime reproduction, as also reported by [54]. In spite of the genetic and environmental homogeneity of the system, there is considerable variability in lifetime reproduction. The calculated values of 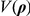 agree well with the observed values, suggesting that there is no need to invoke heterogeneity to explain the variance. The observed skewness is slightly negative, and is underestimated by the calculated values of 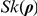.

The three reward models: one using the empirical moments of age-specific reproduction, one using Poisson moments, and one treating age-specific reproduction as fixed, give very different results. The Poisson and the fixed rewards models seriously underestimate the variance and exaggerate the negative skewness of lifetime rewards. In this case, the variability in stage-specific rewards cannot be ignored. That variance is considerably larger than the Poisson expectation, with an index of dispersion (variance-to-mean ratio) of 10.3, 7.1, and 5.8 for the three genotypes. With sample sizes of 800, 800, and 1000, respectively, the variance is greater than Poisson at a significance level too small to be calculated.

The negative skewness in lifetime reproduction seems to arise from a combination of high survival through reproductive life and low variability in reproduction for the survivors. The distributions generated by the Poisson or fixed rewards models contain a small peak at zero (the rare individuals who died before reproducing) and a large peak centered around the mean reproductive output (simulation data, not shown here).

**Figure 2:**
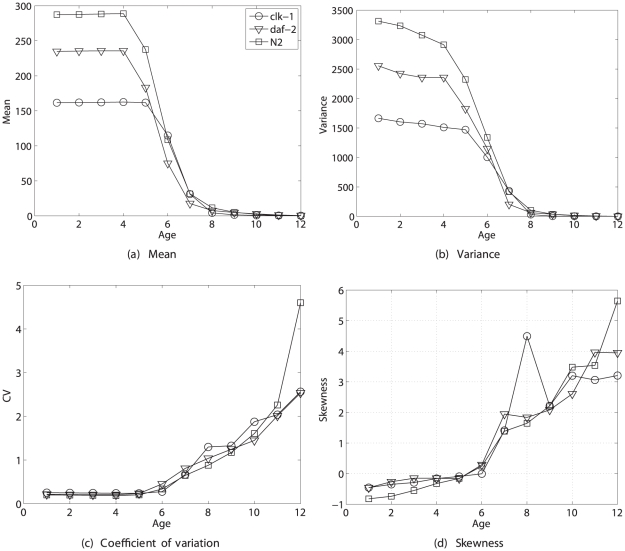
The statistics (mean, variance, coefficient of variation, and skewness) of remaining lifetime reproductive output as a function of age, for the nematode *Caenorhabditis elegans*. Results are shown for two longevity mutants (*clk-1* and *daf-2*) and the standard laboratory strain (*N2*), based on calculations form the empirical moments of rewards. Based on data from [54].

Figure 1 shows statistics of lifetime reproduction from birth, but the reward vector 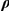 also contains information on the remaining lifetime reproductive output of individuals of any age. Figure 2 shows the mean, variance, coefficient of variation, and skewness of remaining reproduction as a function of age. Mean reproduction declines with age as individuals pass through the reproductive age classes. The variance declines, but the relative variability, as measured by the CV, and the skewness both increase with age.

### Case study 2: Pollutant responses in the polychaete *Streblospio benedicti*

*Streblospio benedicti* is a deposit-feeding spionid polychaete common in estuarine, salt marsh, and shallow subtidal habitats, where it is frequently exposed to various pollutants. Levin et al. [55], [56] conducted a laboratory experiment to measure the demographic effects of exposure to sewage sludge, fuel oil, and blue-green algae. Life expectancy in the laboratory was 20–50 weeks, depending on conditions. Reproduction is sexual, with embryos retained within the body of the female for 4–5 days, before being released as planktonic larvae.

The algae and oil treatments significantly reduced reproduction and population growth rate compared to controls [55]. Population growth rate in the sewage treatment was not significantly different from the control, and indeed, *S. benedicti* is so tolerant of increased nutrient levels that it is often used as an indicator of anthropogenic nutrient input [56].

Laboratory cultures in this study were developed from worms collected from the field, and hence are presumably genetically heterogeneous, certainly more so than is the case for *C. elegans*.

#### Study design

Individual survival and fertility (number of larvae released) were recorded for cohorts under control and three exposure treatments. Data were available for the complete distribution of age-specific reproduction, and for the distribution of total lifetime reproduction.

#### Demographic model

Demography was described with an age-classified projection matrix with projection interval of 1 week.

#### Reproductive rewards

Larval production was measured for each individual at each week; hence
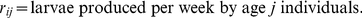
(55)Rewards are described by their empirical moments, by the Poisson model, and by the fixed reward model.

#### Results

**Figure 3:**
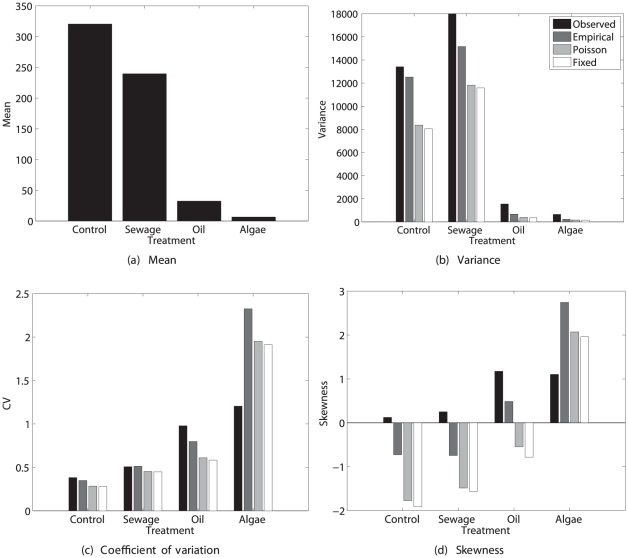
Statistics (mean, variance, coefficient of variation, and skewness) of lifetime reproductive output of the polychaete *Streblospio benedicti* under four pollutant manipulations (control, sewage, fuel oil, and blue-green algae). Results are shown for the observed statistics of lifetime reproduction and the values calculated from the empirical moments, the Poisson reward model, and the fixed reward model. Based on data from [55].

Figure 3 shows the statistics of lifetime reproduction. Mean lifetime rewards were slightly reduced in the sewage treatment, and dramatically reduced in the oil and blue-green algae treatments. There is considerable variation around this mean; on an absolute scale, variances are much higher in the control and sewage treatment than in the oil and algae treatments. On a relative scale, the pattern is reversed; the CV of lifetime reproduction increases from control through sewage and oil treaments, to the algae treatment. The 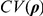 for *S. benedicti* is about 2–5 times greater than that for *C. elegans*. The calculated skewness values increase from control to the algae treatment. Except for the algae treatment, there is a consistent pattern of the observed skewness (which includes effects of unobserved heterogeneity) being more positive than the calculated skewness (which excludes heterogeneity).

The differences among the full distribution, Poisson, and fixed reward models are relatively small. Except in the algae treatment, the calculated variance is smaller than the observed variance, and the calculated skewness less than the observed skewness. This may be a consequence of heterogeneity, if increasingly stressful treatments reveal more effects of such heterogeneity.

**Figure 4:**
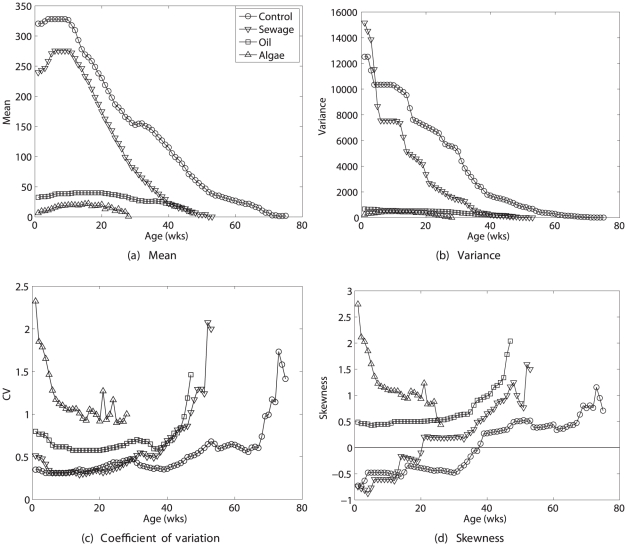
The statistics (mean, variance, coefficient of variation, and skewness) of remaining lifetime reproductive output as a function of age, for the polychaete *Streblospio benedicti*, under four pollutant manipulations (control, sewage, fuel oil, and blue-green algae). Calculated using the empirical moments of age-specific reproduction. Based on data from [55].

Figure 4 shows the statistics of remaining lifetime reproduction as a function of age. Mean lifetime reproduction first increases and then decreases with age; the variance declines dramatically with age. The relative variability, as measured by the 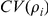 increases with age for all treatments except the algae treatment. The skewness increases with age, again except for the algae exposure treatment. The CV and the skewness decline with age for the algae treatment, suggesting that extreme demographic stress can change what appears to be a typical pattern for less stressful conditions.

### Case study 3: Historical changes in the human population of Sweden

Both *C. elegans* and *S. benedicti* produce large numbers of offspring. The distributiion of stage-specific rewards will be different for a monovular species (producing only a single offspring). If multiple births are ignored (they account for approximately 1% of live births), humans fall into this category. As an example, I analyze a historical sequence of mortality and fertility for the human population of Sweden from 1891 to 2007 [57],[58]. This period included two world wars, the 1916 flu epidemic, and a health transition sufficient to raise female life expectancy at birth from 53 to 83 years.

#### Study design

The data are cross-sectional measurements of age-specific mortality and fertility. In the absence of individual longitudinal data, the distribution of lifetime reproductive output is not available.

#### Demographic model

Rewards were analyzed using an age-classified projection matrix with an age interval and projection interval of 1 year.

#### Reproductive rewards

Rewards are defined as female births
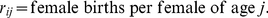
(56)Rewards were described by the Bernoulli model and the fixed reward model. Given the neglect of multiple births, the Bernoulli model gives the actual moments of births. The fixed reward model, in this case, transforms a situation in which a proportion 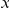 of women give birth to one in which every woman gives birth to a fraction 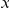 of a child.

#### Results

**Figure 5:**
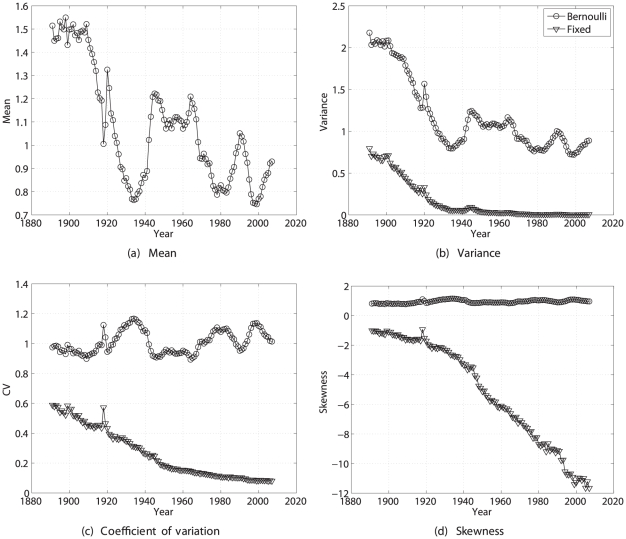
The statistics (mean, variance, coefficient of variation, and skewness) of lifetime reproductive output of Swedish women from 1891 to 2007. Results are shown for the Bernoulli reward model and the fixed reward model.

The statistics of lifetime reproductive output are shown as a function of time in Figure 5. Mean lifetime reproduction fluctuated around 1.5 from 1890 to about 1915, and then declined dramatically until the early 1930's. This was followed by an increase to a period with 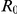 above replacement level (early 1940s to early 1960s), and then another decline. As in many developed countries, mean lifetime reproduction has been below replacement level since the 1970s. The variance calculated from the Bernoulli model declined over that time period, but the CV fluctuated around 1 with no clear trend. The skewness remained roughly constant at about 1. The fixed reward model gives very different results; it shows the variance in lifetime reproduction declining to near zero, and the skewness becoming very negative. This reflects the high survival in Sweden in recent years. With almost every woman living through childbearing years, and with fixed age-specific rewards, there is very little variance in lifetime reproductive output. The distribution eventually consists of a small left hand tail of individual who die before completing reproduction, and a peak of individual who live through childbearing years. The result is a negative skew, and since skewness is scaled relative to the standard deviation, the low variance yields a large negative skewness.

**Figure 6:**
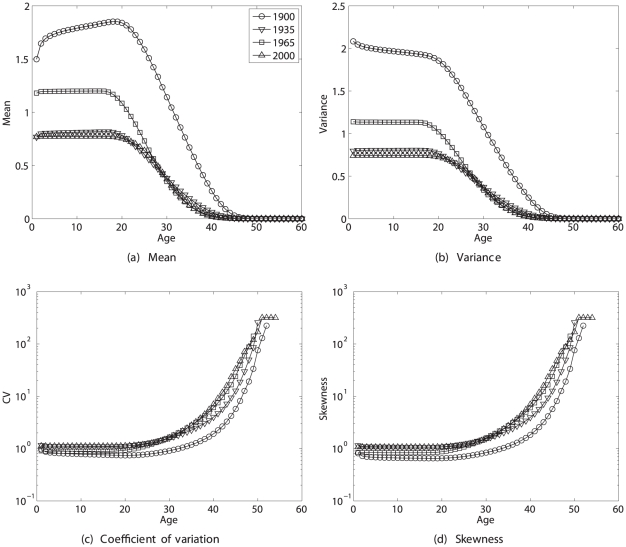
The statistics (mean, variance, coefficient of variation, and skewness) of remaining lifetime reproductive output as a function of age, for Swedish women in 1900, 1935, 1965, and 2000. Calculated using the Bernoulli reward model.

The statistics of remaining lifetime reproduction as a function of age are shown for four selected years in Figure 6. The mean and variance of 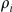 decline with increasing age, whereas 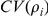 and 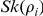 both increase dramatically with age. The patterns of CV and skewness are quite similar, a fact to which I will return in the Discussion.

### Case study 4: Effects of pollen supplementation on *Trillium grandiflorum*

In age-classified populations, an individual either survives to the next age class or dies. Stage-classified models permit a greater diversity of individual trajectories through the life cycle. Thus one might expect individual stochasticity in those trajectories to be more important in determining lifetime reproduction in stage-classified models.

*Trillium grandiflorum* is a perennial herb found in deciduous forests of eastern North America. Knight [59], [60] developed a stage-classified model for *Trillium* with six stages (stage 1 = germinants, 2 = seedlings, 3 = one-leaf plants, 4 = small three-leaf plants, 5 = large three-leaf plants, 6 = reproductive plants). Germinants are newly germinated seeds which remain below ground for their first year of life. At Knight's study sites, *T. grandiflorum* is self-incompatible, and pollinated by bumblebees. To see if reproduction was limited by pollen, she conducted pollen supplementation experiments, which significantly increased seed production [59, Fig. 2]. This manipulation is interesting here because it directly affects the distribution of stage-specific reproductive output without intentional effects on survival.

#### Study design

Demographic data were obtained as a cross-sectional field study. Individual seed production was measured under control and pollen supplementation conditions.

#### Demographic model

Demography was modelled using a stage-classified projection matrix with a projection interval of one year. Transition probabilities under “no herbivory” conditions [59, Fig. 3b] were used to construct the transition matrix 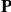


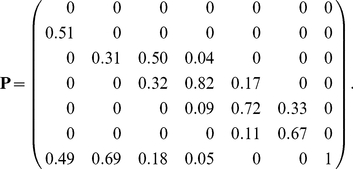
(57)Note that individuals in stages 4–6 may increase or decrease in size, or remain in the same stage.

#### Reproductive rewards

Rewards were defined as seed production. Because only stage 6 reproduces, 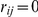 for 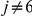, and
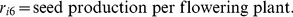
(58)Rewards were measured by their empirical moments (data provided by T. Knight), the Poisson model, and the fixed reward model.

#### Results

**Figure 7:**
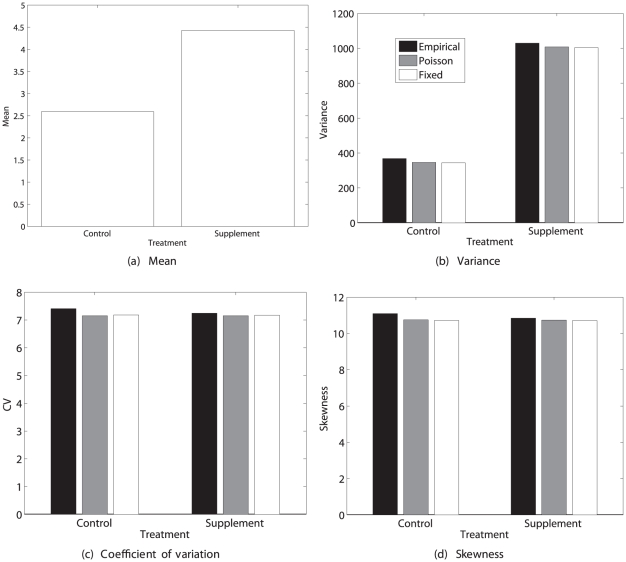
The statistics (mean, variance, coefficient of variation, and skewness) of lifetime reproductive output of the perennial plant *Trillium grandiflorum*, under control and pollen supplementation treatments. Results are calculated from the empirical moments of rewards, the Poisson reward model, and the fixed reward model. Data from [59].

Figure 7 shows the statistics of lifetime reproduction for an newly germinated seed. The pollen supplementation treatment nearly doubled the mean lifetime seed production, and increased the variance by an even greater factor. Relative variability, as measured by the 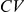, was nearly identical for the control and pollen supplement treatments. The skewness was large, positive (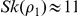) and similar in both treatments.

The Poisson and the fixed reward models produced very similar results, nearly identical to those based on the empirical moments. This contrasts with the results from the age-classified examples, and suggests that much more of the variability in lifetime reproductive output is due to individual stochasticity in this stage-classified model.

**Figure 8:**
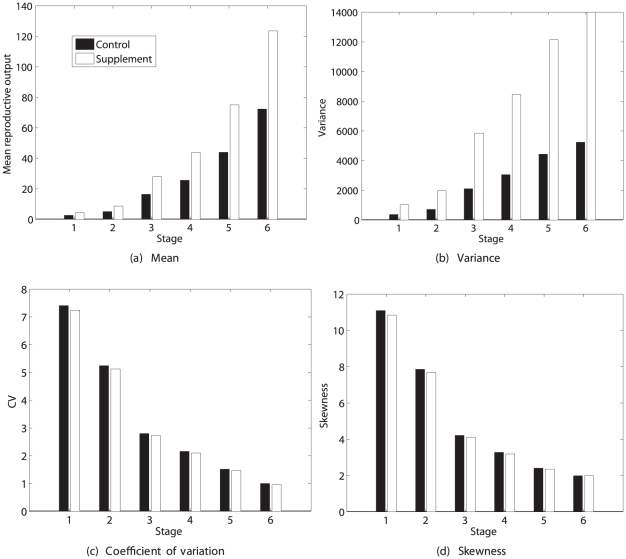
The statistics (mean, variance, coefficient of variation, and skewness) of remaining lifetime reproductive output of stages 1–6 of the perennial plant *Trillium grandiflorum* under control and pollen supplementation treatments. Calculated from the empirical moments of rewards, based on data from [59].

The remaining lifetime reproductive output as a function of individual stage is shown in Figure 8. Mean lifetime reproduction increases with increasing plant size, as does the variance. The *CV* decreases from about 7 for germinants to about 1 for flowering plants. The skewness is positive, declining from about 11 for germinants to about 2 for flowering plants.

### Case study 5: *Lobularia maritima* in a seasonal environment

Species that live in strongly seasonal environments often exhibit reproductive output coupled to the periodic seasonal signal. This is the case for *Lobularia maritima*, an herbaceous perennial crucifer distributed around the Mediterranean basin [61]. The Mediterranean climate is strongly seasonal (cold winters and hot dry summers), and *L. maritima* has an unusually extended 10-month flowering season. Picó et al. [61] developed a periodic model for this plant; here I will analyze lifetime reproduction using the time-varying analysis described in the section *Analysis*.

#### Study design

Cross-sectional data were collected by in a field study, in which the year was divided into 6 periods of 2 months each [61]. Published results include only the mean reproductive output per individual. Two types of reproduction were defined: production of seeds, and production of seedlings.

#### Demographic model

Pico et al. [61] presented a periodic model, with five stages (1 = seeds, 2 = seedlings, 3 = small adults, 4 = medium adults, 5 = large adults). Matrices were reported for September, November, January, March, May, and July. The model is thus a stage-classified projection matrix, with a projection interval of 2 months within the year, and 1 year between years. My analyses were based on mean parameter values over a five-year study [61, Table 2]. The periodic model was constructed using (49) for 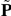 and (50) for the 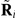, with 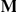 in (49) given by a circulant matrix of the form (40).

#### Reproductive rewards

Adult plants at time 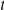 can produce seeds at time 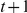 (2 months later), or can produce seeds that germinate to become seedlings at time 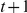. The production of seeds and of seedlings thus constitute two modes of reproduction in this life cycle. Each reproductive mode may exhibit its own pattern of variability, so I have analyzed each of them in order to compare their statistics. Rewards are defined as
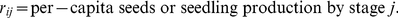
(59)Because only means were reported [61], rewards were described using the Poisson model and the fixed reward model.

#### Results

**Figure 9:**
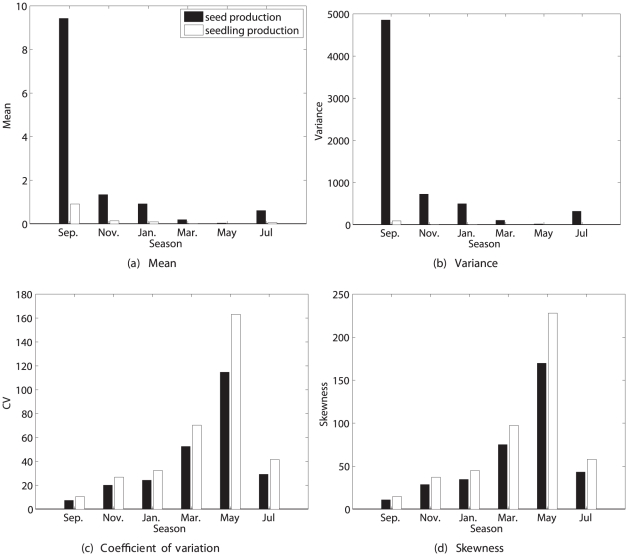
The statistics (mean, variance, coefficient of variation, and skewness) of lifetime reproduction of an individual of the perennial plant *Lobularia maritima*, beginning life as a seed, as a function of the season. Results are shown for reproduction measured as seeds and as seedlings, using the Poisson reward model. Results for the fixed reward model are nearly identical, and not shown. Based on data from [61].

**Figure 10:**
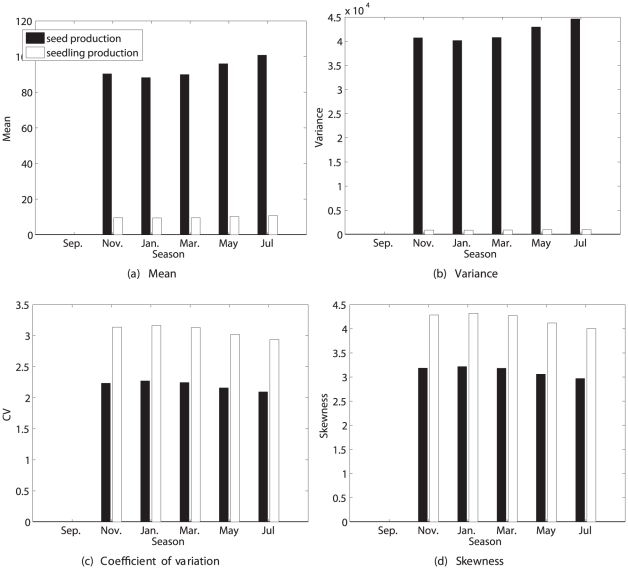
The statistics (mean, variance, coefficient of variation, and skewness) of lifetime reproduction of an individual of the perennial plant *Lobularia maritima*, beginning life as a seedling, as a function of the season. Results are shown for reproduction measured as seeds and as seedlings, using the Poisson reward model. Results for the fixed reward model are nearly identical, and not shown. Based on data from [61].

The prospects for lifetime reproduction by seeds and by seedlings are quite different (Figures 9 and 10). Expected lifetime reproduction of a seed is strongly season-dependent, being high for a seed in September and lower in all other seasons. Expected seed production is much higher than expected production of seedlings. The variance 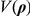 is also high in September and much lower in other seasons, but 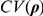 is lowest in September, increasing through May, and is higher for seedling production than for seed production. The 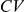 exceeds that of *Trillium* by more than an order of magnitude. Skewness follows a similar pattern, and is extremely large and positive. This level of variance and of positive skewness is implied by the reported vital rates and their seasonal variation, without any contribution from unobserved heterogeneity.

Only results for the Poisson model are shown in Figures 9 and 10; those for the fixed reward model are nearly indistinguishable, implying that almost all the variance in lifetime reproduction is due to individual stochasticity rather than to variance in stage-specific reproduction.

Expected lifetime reproduction for a seedling is zero in September, as are all the moments, because survival of seedlings in September is zero. There is little difference among the other seasons. Mean lifetime seed production is higher than mean seedling production, and the change from seed to seedling increases expected reproduction by an order of magnitude (cf. Figures 9a and 10a). The 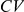 and skewness are higher for seedling production than for seed production. However, the future success of a seedling is much more certain than that of a seed.

These results quantify what might have been expected: that developing to the seedling stage increases the mean, and reduces the variance, of lifetime reproduction. It would be harder to predict a prior the changes in, or the seasonal patterns of, the 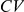 and skewness without an analysis like the one presented here.

### Case study 6: *Lomatium bradshawii* in a stochastic fire environment

*L. bradshawii* is an endangered herbaceous perennial plant now occurs as a few isolated populations in prairies of Oregon and Washington. These habitats were, until recent times, subject to frequent natural and anthropogenic fires. *L. bradshawii* is well adapted to fires, which increase plant size and seedling recruitment, although the effect fades after a few years. Populations that have been recently burned exhibit higher growth rates and lower probabilities of extinction than unburned populations [62]–[65].

A stochastic demographic model for *L. bradshawii* was developed by Caswell and Kaye [63], based on data from an experimental burning study. The study investigated two sites; here I analyze results from one of them (Rose Prairie), in which a critical fire frequency of 0.4–0.5 per year was found to be necessary to maintain the population, the value depending slightly on the autocorrelation of the fire process [63].

#### Study design

Cross-sectional data were collected in different years and at different times since the last experimental fire; these were combined to give stage-classified projection matrices for each state of the fire environment. Only mean reproductive output is available, and because the study did not follow cohorts, no measurements of actual lifetime reproductive output are available.

#### The demographic model

Population dynamics were described with a stage-classified model with stages based on size and reproductive status (stages 1 = seedlings, 2 = small vegetative plants, 3 = large vegetative plants, 4 = small reproductives, 5 = medium reproductives, and 6 = large reproductives). The environment was classified into four states defined by the time since the most recent fire: state 1 = the year of a fire, state 2 = one year post-fire, state 3 = two years post-fire, and state 4 = three or more years post-fire. Projection matrices with a projection interval of one year were derived for each environmental state [63]. The life cycle permits considerable movement among the larger size classes; the matrix 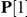, for environmental state 1, for example, is
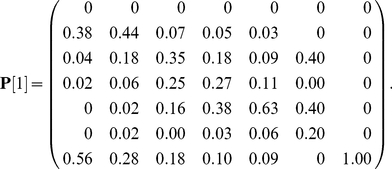
(60)The fire environment is described by a four-state Markov chain. If 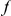 is the long-term frequency of fire, then the transition matrix of the environmental states is
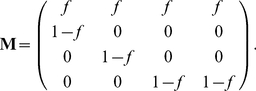
(61)Although fires in this model occur independently from year to year, the environmental states defined in terms of time since fire are autocorrelated; see [63] for details.

#### Rewards

Rewards were measured as production of new seedling plants,
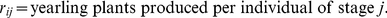
(62)Data on individual reproductive output are not available, so the moments of the rewards were described using the Poisson model and the fixed reward model.

#### Results

**Figure 11:**
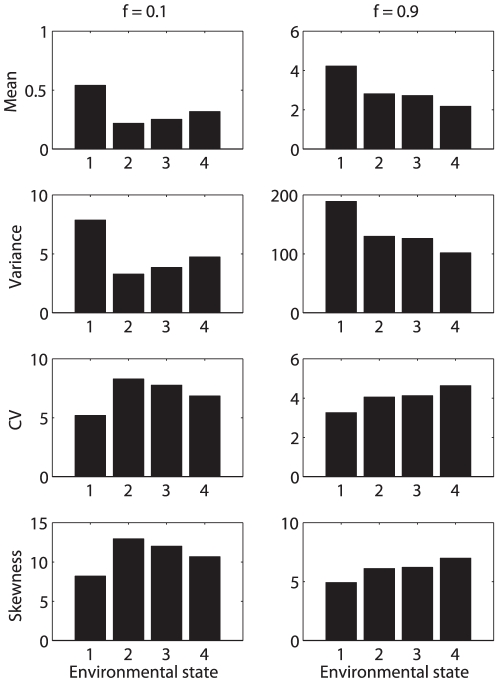
The statistics (mean, variance, CV, and skewness) of lifetime reproduction of the perennial plant *Lomatium bradshawii* in a stochastic fire environment, with fire frequency 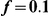
 **and** 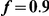 **.** Results are shown as a function of the initial environmental state (state 1 = year of fire, 2 = one year post- fire, 3 = two years post-fire, 4 = three or more years post-fire), calculated using the Poisson reward model. The fixed reward model produces almost identical results. Based on data in [63].

The statistics of lifetime reproduction depend on the initial stage, the initial environmental state, and the fire frequency in the environment. Figure 11 shows the mean, variance, CV, and skewness of lifetime reproductive output for seedlings in each environmental state, at two selected fire frequencies (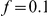 and 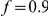). The lower fire frequency reduces the mean and variance, and increases the CV and skewness, compared to the high fire frequency. Individuals in environmental state 1 (the year of a fire) have the highest mean and lowest CV and skewness of lifetime reproduction.

**Figure 12:**
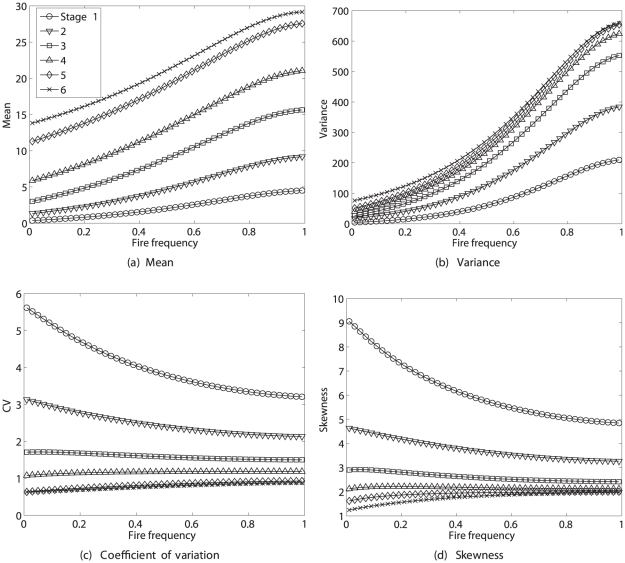
The statistics (mean, variance, CV, and skewness) of remaining lifetime reproduction for each stage of *Lomatium bradshawii* in a stochastic fire environment, as a function of the fire frequency. Values are calculated from moments averaged over the stationary distribution of the environment according to (53). Calculated using the Poisson reward model; results for the fixed reward model are nearly identical. Based on data from [63].

Averaging the moments of 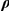 the over the stationary distribution of the environment according to (53) yields the results in Figure 12, as a function of fire frequency. The mean and variance of lifetime reproduction increase with fire frequency and with increasing life cycle stage. The CV and skewness of lifetime reproduction decrease with increasing fire frequency for early stages and increase slightly for later stages. Stages 5 and 6, and to a lesser extent stage 4, converge to a CV of 1 and skewness of 2 as fire frequency increases.

## Discussion

### A calculation protocol

The results in Proposition 1 make it possible to calculate the statistics of lifetime reproductive output implied by a wide array of demographic models: time-invariant or time-varying, age- or stage-structured, and with the reproductive “rewards” defined in a variety of ways. The calculation protocol is:

1. Create the Markov chain transition matrix
  - time-invariant models
    - obtain the transient matrix 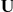

    - construct the transition matrix 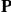 using (3).
  - time-varying models
    - define environmental states 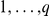

    - obtain environmental state-specific transient matrices 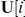, 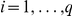

    - construct environmental state-specific transition matrices 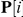, 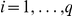, using (3)
    - construct the block-diagonal matrix 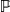 using (43)
    - define the environment transition matrix 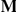 appropriate to the type of environmental dynamics (periodic, stochastic, etc.)
    - construct the block-diagonal matrix 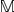 using (45)
    - construct the time-varying transition matrix 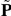, for the process classified jointly by life cycle stage and environmental state, using (49)
2. Create the reward matrices
  - time-invariant models
    - choose a reward measure (eggs, seeds, larvae, seedlings, etc.)
    - specify a reward model[s] (empirical distribution, Poisson distribution, fixed rewards, etc.)
    - create the matrices containing moments of stage-specific rewards, 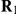, 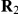, 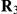

  - time-varying models
    - choose a reward measure
    - specify a reward model[s]
    - construct environmental state-specific matrices of moments of stage-specific rewards, 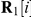, 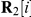, 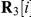, for 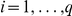

    - construct the block moment matrices 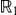, 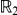, 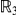 using (48)
    - construct the reward matrices 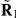, 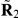, 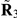, for the the process classified jointly by life cycle stage and environmental state, using (50)
3. Set initial conditions:
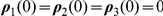

4. Iterate equations (10)–(12) until 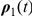, 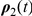, and 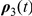 converge to their equilibria.
5. Calculate statistics. Use equations (24)–(27) to compute the variance, standard deviation, CV, and skewness of lifetime reproduction.

These calculations are easily programmed in Matlab or any other matrix-oriented language.

### Data requirements

A Markov chain with rewards requires two kinds of data: the transition matrix 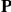 for the Markov chain, and data on the distribution of the rewards 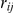 associated with transitions among states of the Markov chain. Transition matrices are available from any demographic study that reports a population projection matrix. The information on reproductive rewards is less standardized. Sometimes reproduction is measured on single individuals; if these data are available, the empirical moments of stage-specific rewards can be used directly. Such data often underlie reported population projection matrices, but only means are usually reported. I encourage researchers with such studies to report the moments of reproduction, or to archive the data so that they can be available for further analysis.

In some cases, reproduction is not measured individually, or offspring cannot be attributed to an individual parent. In such cases, the distribution of rewards can be described with a statistical model; the Poisson, Bernoulli, and fixed reward models used here are examples, but others could be developed. In at least some cases, involving stage-classified populations, the full distribution of stage-specific rewards appears to have little effect on the statistics of lifetime reproductive output.

### Variability and heterogeneity

Variability among individuals in lifetime reproductive output may arise from three sources:

1. Differences among individuals in the pathways taken through the life cycle (individual stochasticity [3] or dynamic heterogeneity [23], [24]).
2. Differences among individuals in the rewards realized at any given stage in the life cycle.
3. Differences among individuals in the transition probabilities 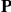 and/or the rewards 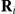.

The variability produced by the first two of these sources arises naturally in any set of individuals experiencing identical vital rates; they are not the result of heterogeneity among individuals. These two sources of variability are incorporated in the Markov chain with reward calculations; hence the statistics calculated from this model provide the desired baseline measurement of variability expected in the absence of heterogeneity.

Source 3, on the other hand, depends on differences among individuals. These may be fixed differences (e.g., genetic differences, or differences in local environment among individuals of sessile species), or differences that develop over time (e.g., accumulated damage caused by environmental factors). Whether fixed or variable, these differences are heterogeneity. Unless they are incorporated into the 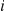-state variables [66] in the demographic model, they are not reflected in the calculations of variability in the Markov chain with rewards.

### Patterns

The examples presented here suggest some interesting patterns that warrant further comparative investigation. A comparison of Figures 1–12 reveals clear differences in the patterns of variability exhibited in these data. Variances can be very large, and skewness can be very positive, simply due to individual stochasticity.

Genetically homogeneous populations of *C. elegans* in constant laboratory conditions exhibit values of 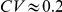 and 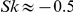. Genetically heterogeneous populations of *S. benedicti* in constant but stressful laboratory conditions exhibit values of 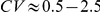 and 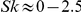. Genetically heterogeneous human populations over a long historical sequence exhibit values of 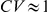 and 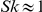. Stage-classified populations of plants (genetically heterogeneous and studied in the field) exhibit higher levels of variability, with 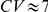 for *T. grandiflorum*, 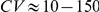 for seed production of *Lobularia maritima*, 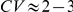 for seedling production of *Lobularia maritima*, and 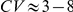 for *Lomatium bradshawii*. Skewness values are similarly more positive, with 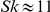 for *T. grandiflorum*, 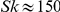 for seed production of *Lobularia maritima*, 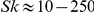 for seedling production of *Lobularia maritima*, and 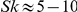 for *Lomatium bradshawii*. It is an open question whether these patterns reflect differences between plants and animals, age-classified and stage-classified models, or field and laboratory conditions.

The studies on *C. elegans* and *S. benedicti* include measurements of observed individual lifetime reproduction (this is one of the important advantages of individual-based studies [67]. This makes it possible to compare the observed variance (which includes the effects of heterogeneity) and the calculated values (which do not).

Tables 1 and 2 show the results, including the standard errors of the empirical variance estimates [68, Section 10.15]. For the *clk-1* and *N2* genotypes of *C. elegans*, the observed variances are well within a single standard error of the variance calculated from the demographic model. For the *daf-2* genotype, the observed variance is about three standard errors less than the calculated value. In the case of *Streblospio benedicti*, the observed variance is greater than the calculated variance in all four treatments. However, because the sample sizes in this experiment were small, the standard errors on the observed variances are too large to say much about these differences. Further studies comparing observed and calculated variances will be useful in detecting heterogeneity in life time reproduction (see also [34]).

**Table 1:** The observed variance in lifetime reproduction and the variance calculated from the demographic model, for three genotypes of *C. elegans*.

*C. elegans*	Genotype		
	*clk-1*	*daf-2*	*N2*
Observed (SE)	1670.3 (83.5)	2211.5 (110.6)	3280.3 (146.7)
Calculated	1665.4	2555.8	3314.4

Standard errors, calculated as in [68], of the observed variances are given in parentheses.

**Table 2:** The observed variance in lifetime reproduction and the variance calculated from the demographic model, for *S. benedicti* under four pollutant exposure treatments.

*S. benedicti*	Treatment			
	Control	Sewage	Oil	Algae
Observed (SE)	14,049 (4335)	19,041 (6530)	1,674 (656)	705 (276)
Calculated	12,519	15,162	671	231

Standard errors, calculated as in [68], of the observed variances are given in parentheses.

In most of the examples, the age or stage patterns of 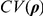 are very similar to the patterns of 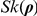. This is expected on the basis of several distributional facts. If lifetime reproduction follows a Poisson distribution, the CV is equal to the skewness. If individuals spend an exponentially distributed length of time in adult reproductive states with a constant reproductive output at each time, the CV is one-half of the skewness. In an age-classified model with high survival and a Bernoulli distribution of numbers of offspring at each age, the lifetime reproduction will have a binomial distribution. If 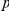 be the probability of success in the Bernoulli trial (i.e., the probability of reproduction), the ratio of the CV to the skewness of a binomial distribution is
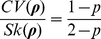
(63)which, if 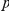 is small, is approximately 1/2. This suggests that age patterns of the CV of lifetime reproduction are probably often similar to the patterns of skewness.

In the cases of *T. grandiflorum* and *L. bradshawii*, the skewness of lifetime reproductive output in adult stages converges to a value close to 2. This value is a kind of benchmark for skewness, in the following sense. Consider a population with the minimum possible variability: all stages have identical survival probability and identical fixed reproductive output. Individual lifetimes are then exponentially distributed, and lifetime reproductive output is proportional to an exponentially distributed random variable. The skewness of this distribution will be the same as that of the exponential distribution, which is 2, regardless of parameter values.

It is interesting to compare the variance from the full reward model (when available), the Poisson reward model, and the fixed reward model. Tables 3 and 4 show the results for the various examples. In the case of *C. elegans*, the Poisson reward model accounts for only 20–30% of the variance in lifetime reproduction. The fixed reward model accounts for only about 10–20%. In the case of *Streblospio benedicti*, also age-classified, the Poisson model accounts for 60–80% of the variance, and fixed model only 1–5 percentage points less. In the case of the human population of Sweden, the proportion of variance explained by the fixed model declines from about 35% in 1891 to nearly 0.

**Table 3:** The fraction of the variance in lifetime reproductive output accounted for by the Poisson reward model and the fixed reward model, relative to the variance calculated from the full empirical moments of the stage-specific rewards.

	Full	Poisson	Fixed
*C. elegans*			
*clk-1*	1.0	0.226	0.130
*daf-2*	1.0	0.206	0.115
*N2*	1.0	0.300	0.213
*S. benedicti*			
control	1.0	0.697	0.643
sewage	1.0	0.779	0.764
oil	1.0	0.584	0.537
algae	1.0	0.706	0.675
*Trillium*			
control	1.0	0.940	0.933
supplement	1.0	0.980	0.975

Results are shown for *Caenorhabditis elegans*, *Streblospio benedicti*, and *Trillium grandiflorum*.

**Table 4:** The fraction of the variance in lifetime reproductive output accounted for by the fixed reward model, relative to the variance calculated from the Bernoulli model, for the human population of Sweden.

Sweden		
Year	Bernoulli	Fixed
1891	1.0	0.365
1911	1.0	0.236
1931	1.0	0.093
1951	1.0	0.036
1971	1.0	0.017
1991	1.0	0.012
2001	1.0	0.006

In the stage-classified examples the situation is quite different. In *Trillium grandiflorum* the Poisson model accounts for 95–98% of the variance, and the fixed rewards model only about 0.5 percentage points less. In the periodic model for *Lobularia maritima* and the stochastic model for *Lomatium bradshawii* the full rewards are not available, so comparisons must be made with the Poisson model. The fixed reward model captures more than 99% of the variance in lifetime reproduction from the Poisson model in *Lobularia* and from 93–98% of the Poisson model variance in *Lomatium* (results not shown).

Analyses of the statistics of lifetime reproductive output in other species, and the responses of those statistics to environmental and demographic differences, will be valuable. The important paper by Steiner and Tuljapurkar [34] takes this one step further and constructs models that include heterogeneity among individuals, and examines the effect of such heterogeneity on the variance in lifetime reproduction. They conclude that heterogeneity will produce only modest changes in the variance.

### Generalizing the concepts of rewards and costs

The results of Proposition 1 can be extended to a wide range of demographic questions by generalizing the concept of reward. For example, a fixed reward model with
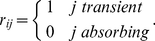
(64)provides a unit reward each time the individual occupies a transient (i.e., living) stage. The lifetime reward is longevity, and Proposition 1 provides a way to calculate all the moments of longevity. These moments can also be computed directly from the fundamental matrix of the absorbing Markov chain [69, Theorem 3.2] [2], [3], [26], but it is useful to have an alternative. Moreover, the reward model (64) can be generalized to include rewards that describe the statistics of longevity weighted by quality of life, health status, income, etc.

### Discounting rewards in ergodic Markov chains

The development of Proposition 1 took advantage of the fact that demographic Markov chains are absorbing, with death as an absorbing state. If the rewards represent reproduction, then rewards in an absorbing state are zero, and since all individuals will eventually be absorbed, the moments 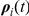 will eventually converge to equilibrium values as rewards stop accumulating.

If the Markov chain is ergodic (or if the dead continue to receive rewards), the situation is different. Rewards will continue to accumulate indefinitely, and accumulated rewards will not converge unless a discount rate is introduced, to value future rewards less than current ones [35]. Let 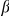 be a discount rate, where 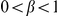. Then the conditional expectation of future rewards in (6) becomes
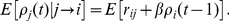
(65)Carrying through the calculations as in Proposition 1 yields
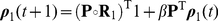
(66)

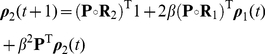
(67)

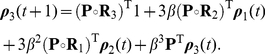
(68)In general, the 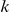
th moments of accumulated rewards are given by
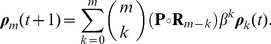
(69)This formulation may have applications in models for, e.g., habitat dynamics where different environmental states are of different value for management or conservation.

### Why 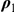 is not 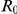


In many cases, the mean lifetime reward 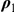 (or its first entry, 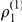) will equal 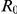. But not always, and it does not share the properties mentioned in Section (measuring the per-generation growth rate and serving as an indicator variable for population growth). 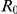 enjoys those properties because it is linked to, and calculated from, a model of population dynamics, either through the familiar age-specific calculation (1), or through the Cushing-Zhou theorem for stage-classified models (2).

The mean lifetime reward 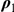 is calculated for a cohort, not a population, and so it has no such linkage. In cases where reproduction is measured in the same currency (eggs, larvae, seeds, etc.) that appears in a population projection matrix, 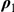 may be numerically equivalent to 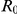. But it is possible, and will often be desirable, to measure reproductive rewards in different currencies, and in such cases 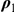 cannot be interpreted as other than what it is: mean lifetime reproductive output, measured in that currency. This is particularly true when the life cycle includes multiple kinds of reproduction. Neither of these alone can serve as the net reproductive rate 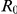. For example, because *Lobularia* has reproductive output measured in seeds and seedlings, the mean lifetime production of these two types of offspring cannot serve as an indicator of population growth. Developing connections to population dynamics is an interesting unsolved problem.
